# Self Dilution of Model Polystyrene Pom‐Poms and Combs by Unentangled Side Arms

**DOI:** 10.1002/marc.202500930

**Published:** 2026-02-10

**Authors:** Max G. Schußmann, Valerian Hirschberg, Manfred Wilhelm, Manfred H. Wagner

**Affiliations:** ^1^ Institute For Chemical Technology and Polymer Chemistry Karlsruhe Institute of Technology (KIT) Karlsruhe Germany; ^2^ Institute of Technical Chemistry Clausthal University of Technology Clausthal‐Zellerfeld Germany; ^3^ Polymer Engineering/Polymer Physics Berlin Institute of Technology (TU Berlin) Berlin Germany

**Keywords:** brittle fracture, polystyrene comb, polystyrene pom‐pom, self dilution, short chain branching, start‐up elongational viscosity

## Abstract

We present linear‐viscoelastic characterization and elongational viscosity start‐up data of 4 model polystyrene pom‐poms and 2 model polystyrene combs. All 6 model systems have a self‐entangled backbone, but unentangled side arms. Pom‐poms consist of a linear backbone with one branching point with q arms at each end of the backbone. Analysis by the Enhanced Relaxation of Stretch (ERS) model shows that the elongational rheology of all model polymers is equivalent to that of polymer melts consisting of long linear chains diluted by short, unentangled linear polymer chains of the same chemistry. Strain hardening increases with increasing intrinsic dilution of the backbone by the arms. The comparison of the elongational viscosity data of pom‐poms versus that of the combs with several branching points along the backbone chain suggests that the location of the unentangled side arms does not result in significant differences. At higher elongation rates, brittle filament fracture is observed, which is well described by the fracture criterion for linear melts and polymer solutions, thus confirming the self‐dilution of the backbone chains of pom‐poms and combs by unentangled side arms.

## Graphical abstract

This study uses experimental rheological data of 4 model polystyrene pom‐poms and 2 model polystyrene combs, all with unentangled arms, and constitutive modelling using Enhanced Relaxation of Stretch (ERS) model to show that the elongational rheology of the branched polymers is equivalent to that of polymer melts consisting of long linear chains diluted by short unentangled linear polymer chain.

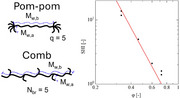

## 1 Introduction

**FIGURE 1:**
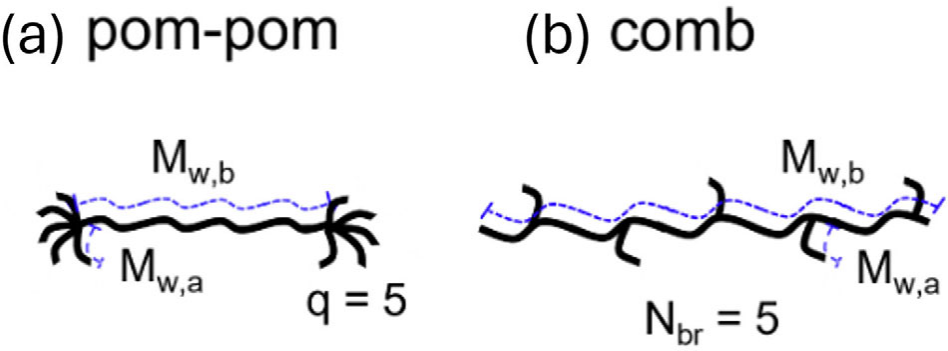
Schematic of (a) pom‐pom polymer: Two point‐like branching sections with q side arms of similar molecular weight M*_w,a_* are connected by a linear backbone chain of molecular weight M*_w,b_*; (b) comb polymer: A linear backbone chain of molecular weight M*_w,b_* with N_br_ side arms of similar molecular weight M*_w,a_*.

A pom‐pom polymer consists of a backbone chain of molecular weight *M_w,b_* with *q* arms of molecular weight *M_w,a_* at both ends of the backbone (Figure 1a) [1, 2, 3]. Originally, the pom‐pom molecule was a theoretical concept simplifying complex polymer topologies and relaxation dynamics with many different kinds of branches, as, e.g., in low‐density polyethene (LDPE), but due to progress in living anionic polymerization, well‐defined pom‐pom polymers can now be synthesised in quantities large enough for extensive rheological characterisation [4, 5]. In comb polymers, by most synthetic approaches, *N_br_* side arms with a molecular weight *M_w,a_* are statistically distributed along the backbone chain of molecular weight *M_w,b_* (Figure 1b)

Intensive rheological characterization in the linear and non‐linear regimes, both in shear and uniaxial elongation of comb and pom‐pom melts, has led to a deeper understanding of the dynamics of these branched systems. Theoretically established concepts explaining the relaxation behaviour of branched polymers, such as hierarchical relaxation and convective constraint release, have been validated against experimental data in the linear and nonlinear viscoelastic regime [6, 7, 8, 9, 10, 11, 12, 13, 14, 15, 16, 17, 18]. Research has been mainly focused on the effects of long‐chain branching, i.e., on systems with molecular weight of the side arms *M_w,a_* being larger than the entanglement molecular weight *M_e_* [1, 19]. Although extensive research has been conducted on long‐chain branches of model polymers and on long (LCB) and short‐chain branching (SCB) in low‐density polyethene, a precise distinction or crossover from rheologically short and long branches in terms of the number of entanglements has not been established so far, to the best of our knowledge. While spectroscopic methods, e.g. ^13^C NMR, will define side carbon lengths larger than 8 CH_2_ and in rare cases up to 20 CH_2_ as LCB, these numbers of CH_2_ groups are substantially below typical entanglement lengths. Consequently, the rheological crossover between SCB and LCB is experimentally not easy to determine due to the lack of model systems and spectroscopic identification. Schußmann et al. [20]. considered six PS pom‐pom melts in shear and elongational flow and distinguished different archetype topologies ranging from (I) pom‐poms with an unentangled backbone and entangled side arms, (II) entangled backbone and entangled side arms, as well as (III) pom‐poms with an entangled backbone and weakly or unentangled side arms.

In the presented work, we focus on linear‐viscoelastic characterization and elongational viscosity start‐up data of 4 polystyrene pom‐poms and 2 polystyrene combs with an entangled backbone, but unentangled side arms. Our analysis shows that pom‐poms and combs with unentangled side arms can be considered as solutions of quasi‐linear polymers, consisting of backbone chains which are diluted by the side arms. This confirms the earlier reported concept of self‐dilution of poly(***α***‐olefin) bottlebrushes by alkane side chains [21, 22] or for Poly(alkyl methacrylate) samples [23, 24], explaining their strain hardening in extensional flow. We also note that Liu et al. [25]. investigated the solvent effect of the short arm of entangled asymmetric three‐arm star polystyrenes. They demonstrated that the effect of the short arm on the plateau modulus is equivalent to the effect of short chains in binary blends consisting of linear long and short chains with equivalent weight fractions as in the asymmetric stars. Earlier work of Wagner et al. [26]. showed that for an asymmetric star with an arm length of about one entanglement, the arm acts as a diluent on the time scale of the backbone, and the elongational rheology of the star is equivalent to that of a linear polymer diluted by short unentangled linear chains.

### 1.1 Materials Characterization and Experimental Methods

As detailed in Ref [4, 5], the pom‐pom polymers were synthesized by the following 2‐step procedure: (1) A Poly(2‐vinylpyridine)‐*b*‐Polystyrene‐*b*‐Poly(2‐vinylpyridine) triblock copolymer backbone is synthesized with a short Poly(2‐vinylpyridine) end block. (2) The precursor arms are polymerized separately and are grafted onto the backbone end group. The molecular parameters of the pom‐poms are listed in Table 1 using the nomenclature $M_{w,b}-$ 2x $q-M_{w,a}$ with molecular weights given in kg/mol.

**TABLE 1:** Molecular parameters of the PS pom‐pom denoted as $M_{w,b}$‐2xq‐$M_{w,a}$. M_w,b_ is the molecular weight of the backbone, q is the number of arms per end group, and $M_{w,a}$ is the molecular weight of the arm. The dispersity of the backbone, arms, and total pom‐pom are Đ_b,_ Đ_a_ and Đ_t_, respectively. The number of entanglements of the backbone $Z_{b}$ and the arms $Z_{a}$ is calculated using an entanglement molecular weight of $M_{e}$ = 16.8 kg/mol [27]. $\varphi_{b}$ is the volume fraction of the backbone. The effective number $Z_{b,\mathrm{eff}}$ of backbone entanglements is calculated by $Z_{b,\mathrm{eff}}=Z_{b}\varphi_{b}$.

Sample	$M_{w,\;b\;}$ [kg/mol]	$Z_{b}$	*Đ*_b_	$M_{w,\;a}$ [kg/mol]	$Z_{a}$	*Đ*_a_	q	$\varphi_{b}$	*Đ*_t_	$Z_{b,eff}$
300k‐2 × 15‐2.5k	300	18	1.40	2.5	0.15	1.06	15	0.80	1.40	14
300k‐2 × 14‐4k	300	18	1.40	4	0.24	1.05	14	0.73	1.40	13
300k‐2 × 9‐8k	300	18	1.40	8	0.48	1.04	9	0.68	1.40	12
280k‐2 × 30‐7k	280	17	1.04	7	0.42	1.03	30	0.40	1.16	7

The PS combs were synthesized by a combination of living anionic polymerization and grafting onto. PS anions (*M_w,a_* = 6 kg/mol) as arms were grafted onto a post‐functionalized PS backbone (*M_w,b_* = 290 kg/mol), containing acetyl groups, which resulted in the short‐chain branched comb topology. A more detailed explanation of this synthesis route and the molecular characterization with SEC‐MALLS and NMR of the comb polymers synthesized by the same route can be found in Abbasi et al. [28, 29, 30]. PS290k is a linear polystyrene melt without any side chains (*M_w_* = 290 kg/mol, *Đ* = 1.10) and used for comparison.

All small‐amplitude oscillatory shear (SAOS) measurements were performed on a TA Instruments ARES G2 rheometer under a nitrogen inert gas atmosphere. A 13 mm parallel plate geometry was used, and angular frequency sweeps typically between 0.01 and 100 rad/s were conducted in the linear regime at temperatures between 125°C and 220°C. Mastercurves of storage modulus *G′* and loss modulus *G*″ at reference temperature 140°C were obtained by time‐temperature superposition (TTS). Elongational measurements were conducted using an extensional viscosity fixture (EVF) with Hencky strain rates of $\dot{\varepsilon }$ = 0.01–10 s^−1^ and temperatures between *T* = 130–180°C up to a maximum Hencky strain of ε = 4 or sample rupture, and data were shifted by TTS to the reference temperature of 140°C.

Dynamic scanning calorimetry (DSC) measurements at 10 K/min for the samples 300k‐2×15‐2.5k and 300k‐2×14‐4k showed glass transition temperatures of 105.5°C and 106.3°C, respectively. Thus, no significant change between the glass transition temperature of pom‐poms with very short branches and high molecular weight linear PS polymers was found, which is in agreement with previous studies by van Ruymbeke et al. [6].

### 1.2 The Enhanced Relaxation of Stretch (ERS) Model

In the following, we give a short summary of the basic equations of the Enhanced Relaxation of Stretch (ERS) model. For details, please see the original publication [31]. We model pom‐poms and combs with an entangled backbone, but unentangled side arms as linear melts consisting of the backbone chains, which are permanently diluted by the side arms, in a similar way as a linear polymer melt is diluted by short, unentangled linear polymer chains of the same chemistry [22]. The extra stress tensor σ is given by

(1) $$\sigma \left(t\right)=\int_{-\infty }^{t}\frac{\partial G\left(t-t^{\prime }\right)}{\partial t^{\prime }}\;f^{2}\left(t,t^{\prime }\right)S_{DE}^{IA}\left(t,t^{\prime }\right)dt^{\prime }$$

The relaxation modulus *G(t)* is expressed by a spectrum of Maxwell modes with relaxation moduli *g_i_* and relaxation times *τ_i_*,

(2) $$G\left(t\right)=\sum_{i}G_{i}\left(t\right)=\sum_{i}g_{i}\exp\left(-t/\tau_{i}\right)\;$$

$S_{\mathrm{DE}}^{\mathrm{IA}}$ is the Doi and Edwards [32, 33] strain tensor assuming independent alignment (IA) of tube segments with $S_{\mathrm{DE}}^{\mathrm{IA}}=5S$, where ***S*** is the second‐order orientation tensor. The molecular stress function *f(t,t’)* is inversely proportional to the tube diameters *a*, and is a function of both the observation time *t* (the time when the stress is measured) and the time *t’* of creation of tube segments by diffusion. If *f(t,t’)* ≡ 1, Equation (1) reduces to the Doi‐Edwards (DEIA) model.

The Molecular Stress Function *f(t,t’)* Is Obtained by the Evolution of the stretch equation.

(3) $$\begin{array}{c} \frac{\partial f\left(t,t^{\prime }\right)}{\partial t}=f\left(K\left(t\right):S\left(t,t^{\prime }\right)\right)-\frac{f-1}{\tau_{R}}\left(1-\varphi^{4}\right)-\frac{\left(f^{5}-1\right)\varphi^{4}}{5\tau_{R}} \end{array}$$

K is the deformation gradient and φ the volume fraction of the polymer in the solution. Compared to the corresponding undiluted linear polymer melt, the stretch function *f* of the solution is reduced by a factor $\varphi^{1/2}$, because the tube diameter increases by a factor $\varphi^{-1/2}$ [34, 35, 36]. This dilution effect is taken into account by Equation (3). For linear polymer melts and solutions of linear polymers, the Rouse stretch relaxation time $\tau_{R}$ can be obtained by the relation

(4) $$\tau_{R}=\frac{12M_{w,b}\;\eta_{0}}{\pi^{2}\rho \;\mathrm{RT}\varphi }\;{\left(\frac{M_{\mathrm{cm}}}{M_{w,b}\varphi }\right)}^{2.4}$$

with ρ the density (in kg/m^3^), *R* the universal gas constant and *T* the absolute temperature, and the volume fraction φ of the backbone chains takes into account backbone dilution by the unentangled side arms [37, 38]. The critical molecular weight *M_cm_* of PS melt is taken as *M_cm_* = 35 kg mol^−1^ equivalent to 2.1 M_e._ [39].

From Equations (1) and (3) and in the limit of high elongation rates, the steady‐state elongational stress $\sigma_{E}$ can be approximated by

(5) $$\sigma_{E}≅5G_{N\;}^{0}f^{2}≅5G_{N\;}^{0}\varphi^{-2}\;\sqrt{5Wi_{R}}=5G_{N,\mathrm{PS}\;}^{0}\sqrt{5Wi_{R}}$$

$G_{N}^{0}$ is the plateau modulus of the diluted backbone chains, $G_{N,\mathrm{PS}\;}^{0}$ the plateau modulus of the neat undiluted polystyrene, and $Wi_{R}=\dot{\varepsilon }\;\tau_{R}$ the Weissenberg number. Thus, the steady‐state elongational stress $\sigma_{E}$ is expected to be independent of the amount of dilution and to depend only on the plateau modulus of the undiluted polymer and the Weissenberg number. Equation (5) is equivalent to the “universal relation” reported by Narimissa et al. [40]. For later use, we rewrite the universal relation in terms of the steady‐state elongation viscosity $\eta_{E}$,

(6) $$\frac{\eta_{E\;}}{\eta_{0}}\;≅\;\frac{5\sqrt{5}G_{N,\mathrm{PS}\;}^{0}\tau_{R}}{\eta_{0}}{\mathrm{Wi}}_{R}^{-1/2}$$

In elongational flow of polymer samples showing strong transient strain hardening, brittle or elastic fracture at higher strain rates is frequently observed [41, 42, 43, 44]. According to the entropic fracture hypothesis, a fracture will occur when the strain energy of a chain segment reaches the bond energy *U* of a carbon‐carbon bond [45, 46]. The fracture criterion for the critical stretch $f_{c}$ at fracture is given by:

(7) $$\varphi \;f_{c}^{2}=\frac{U}{3kT}\;\varphi$$

with *k* being the Boltzmann constant and *T* the absolute temperature. The ratio of bond‐dissociation energy *U* to thermal energy *3kT* is *U/3kT* = 34 at a temperature of *T* = 140°C. From Equation (5) with $\;f^{2}={\;f}_{c}^{2}$ and $Wi=Wi_{RC}={\dot{\varepsilon }}_{c}\;\tau_{R}$, the critical elongational fracture stress $\sigma_{c}$ at the critical Weissenberg number $Wi_{\mathrm{Rc}}$ is given by:

(8)

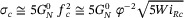




From Equation (7) and the First Identity of Equation (8), We Obtain

(9) $$\frac{\sigma_{c}\varphi }{G_{N}^{0}}≅5\frac{U}{3kT}≅170$$

and from the second identity of Equation (8)

(10) $$\frac{Wi_{Rc}}{\varphi^{2}}≅\frac{1}{5}{\left(\frac{U}{3kT}\right)}^{2}≅231$$

Within experimental accuracy, the validity of Equations (8) and (9) has been verified by comparison to fracture data of linear PS melts and solutions [46].

## 2 Results and Discussion

The mastercurves of storage modulus *G*′ and loss modulus *G*″ were fitted with about ten relaxation modes using the IRIS software [47, 48]. From the first and second moments of the parsimonious relaxation time spectrum, the zero‐shear viscosity $\eta_{0}$, the viscosity average relaxation time $\tau_{v}$, the disengagement time $\tau_{d}$ and the steady‐state compliance $J_{s}^{0}$ are obtained by:

(11) $$\eta_{0}=\sum_{i}g_{i}\tau_{i}$$

(12) $$\tau_{v}=\frac{\eta_{0}}{G_{N}^{0}}\;$$

(13) $$\tau_{d}=\frac{\sum_{i}g_{i}\tau_{i}^{2}}{\eta_{0}}\;$$

(14) $$J_{s}^{0}=\frac{\sum_{i}g_{i}\tau_{i}^{2}}{\eta_{0}^{2}}\;$$

$\tau_{d}$ is a representative measure of the long‐time part of the relaxation time spectrum, and $J_{s}^{0}$ determines the recoverable deformation or “elasticity” in the LVE regime. By considering the identity $J_{s}^{0}\;G_{N}^{0}=\tau_{d}/\tau_{v}$, it is also a measure of the broadness of the relaxation time spectrum.

While $\varphi_{b}$ is the volume fraction of the backbone in the polymer as calculated from the stoichiometry of the polymers as obtained from the GPC analysis, and as given in Tables 1 and 2, φ is calculated from the rheological data

(15) $$\phi =\sqrt{G_{N}^{0}/G_{N,PS}^{0}}$$

with $G_{N,\mathrm{PS}}^{0}$ being the plateau modulus of neat (undiluted) linear PS taken as $G_{N,PS}^{0}≅250\;kPa$. [49] As seen from Table 3, the presence of residual precursor side arms, which are not attached to the backbone, as well as possible traces of residual solvent, ϕ is always less than or equal to $\varphi_{b}$. As ϕ rather than $\varphi_{b}$ represents the real effective rheological dilution of the backbone through the rheological measurement of the plateau modulus and the diluted modulus, and calculation through Equation (14), which we will use ϕ in the analysis of the elongational viscosity start‐up data in the following. The Rouse time $\tau_{R}$ is calculated from Equation (4) with molar mass *M = M_w,b_* and backbone fraction φ.

**TABLE 2:** Molecular characteristics of PS combs denoted as *Mw,b – N_br_ – Mw,a. M_w,b_* is the molecular weight of the backbone, $N_{\mathrm{br}}$ is the number of side arms, and $M_{w,a}$ is the molecular weight of the side arm. The number of entanglements of the backbone $Z_{b}$ and the arms $Z_{a}$ is calculated using an entanglement molecular weight of $M_{e}$ = 16.8 kg/mol [27]. The dispersity of the total comb is *Đ_t._* The effective number $Z_{b,\mathrm{eff}}$ of backbone entanglements is calculated by $Z_{b,\mathrm{eff}}=Z_{b}\varphi_{b\;}$.

Combs	$M_{w,b}$ [kg/mol]	$Z_{b}$	*Đ*_b_	$N_{br}$	$M_{w,a}$[kg/mol]	$Z_{a}$	*Đ*_a_	$M_{w}$ [kg/mol]	$\varphi_{b}$	*Đ*_t_	$Z_{b,eff}$
PS290k	290	17	—	—	—	—	—	290	1.00	1.10	17
290k‐50‐6k	290	17	1.10	50	6	0.36	1.04	750	0.49	1.09	8
290k‐100‐6k	290	17	1.10	100	6	0.36	1.06	900	0.33	1.07	6

**TABLE 3:** Rheological characterization of PS pom‐poms at 140°C: Plateau modulus $G_{N}^{0}$, zero‐shear viscosity $\eta_{0}$, disengagement time $\tau_{d}$, Rouse stretch relaxation time $\tau_{R}$, and steady‐state compliance $J_{s}^{0}$.

Sample	$G_{N}^{0}$ [kPa]	φ [‐]	$\varphi_{b}$ [‐]	$\eta_{0}$ [MPa s]	$\tau_{d}$ [s]	$\tau_{R}$ [s]	$J_{s}^{0}$ [kPa^−1^]
300k‐2 × 15‐2.5k	130	0.72	0.80	67^*^	1.2^:^10^4^	125	0.27
300k‐2 × 14‐4k	130	0.72	0.73	15	3.7^:^10^3^	27	0.26
300k‐2 × 9‐8k	91	0.60	0.68	60	2.8^:^10^4^	206	0.46
280k‐2 × 30‐7k	28	0.33	0.40	1.2	2.4^:^10^2^	34	0.19
PS290k	230	0.96	1.00	160	2.9^:^10^3^	119	0.02
290k‐50‐6k	52	0.46	0.49	39	1.8^:^10^4^	365	0.46
290k‐100‐6k	28	0.33	0.33	22	1.8^:^10^4^	592	0.82

* Extrapolated (see text)

In Table 3, the measured plateau modulus $G_{N}^{0}$, backbone volume fractions φ and $\varphi_{b}$, zero‐shear viscosity $\eta_{0}$, disengagement time $\tau_{d}$, Rouse stretch relaxation time $\tau_{R}$, and the steady‐state compliance $J_{s}^{0}$ are summarized.

**FIGURE 2:**
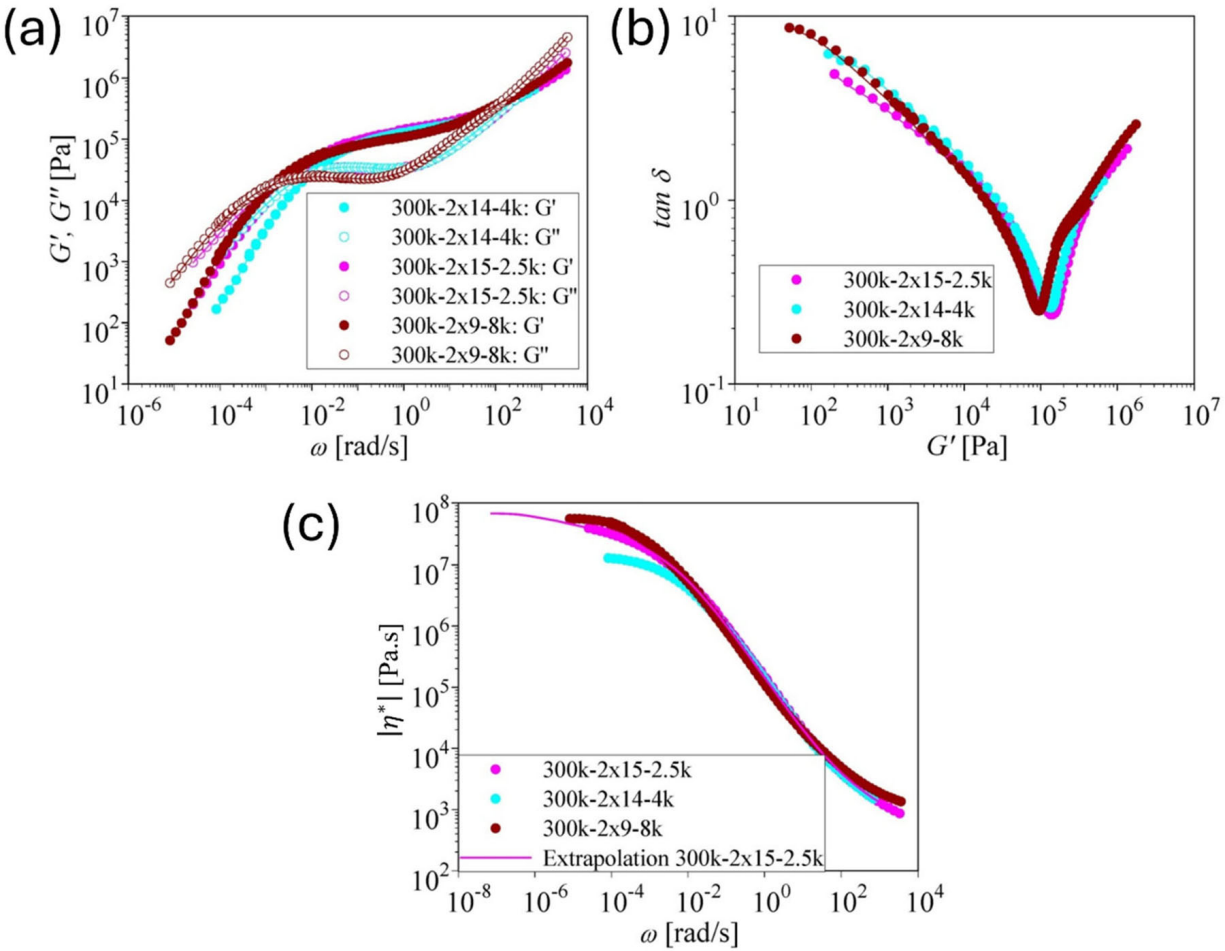
(a) Storage modulus *G*′ and loss modulus *G*″ as a function of frequency *ω* for pom‐poms 300k‐2×15‐2.5k, 300k‐2×14‐4k, and 300k‐2×14‐8k. (b) Loss tangent δ as a function of *G*′. (c) Complex shear viscosity $|\eta^{*}\left|\left(\omega \right)\right|$.

We first consider the shear and elongational rheology of the pom‐pom series with an entangled backbone of M_w,b_ = 300 kg/mol and unentangled side arms with M_w,a_ = 2.5, 4, and 8 kg/mol (Table 1). Figure 2 shows the master curves of storage modulus G′ and loss modulus G″ as a function of angular frequency ω, the loss tangent δ as a function of G′, and the absolute value of the complex shear viscosity |$\eta^{*}\left(\omega \right)$ |. All three pom‐poms feature one minimum with tanδ<1, indicating the rubber plateau $G_{N}^{0}$ created by the entangled backbones. The plateau modulus, $G_{N}^{0}$, is taken as the value of G′ at the minimum of tanδ and is given in Table 3. Due to the higher dilution by the unentangled side arms and therefore lower volume fraction of the backbone, the plateau modulus of pom‐pom 300k‐2×9‐8k is significantly lower than for 300k‐2×15‐2.5k and 300k‐2×14‐4k. For pom‐pom 300k‐2×15‐2.5k the terminal regime is not fully resolved by SAOS, and therefore the zero‐shear viscosity had to be extrapolated by use of the Carreau‐Yasuda model. $\eta^{*}=\frac{\eta_{0}}{1+{\left(\beta \omega \right)}^{c}}$ (Figure 2c and Table 3) to obtain a valid estimation of the Rouse time from Equation (4).

**FIGURE 3:**
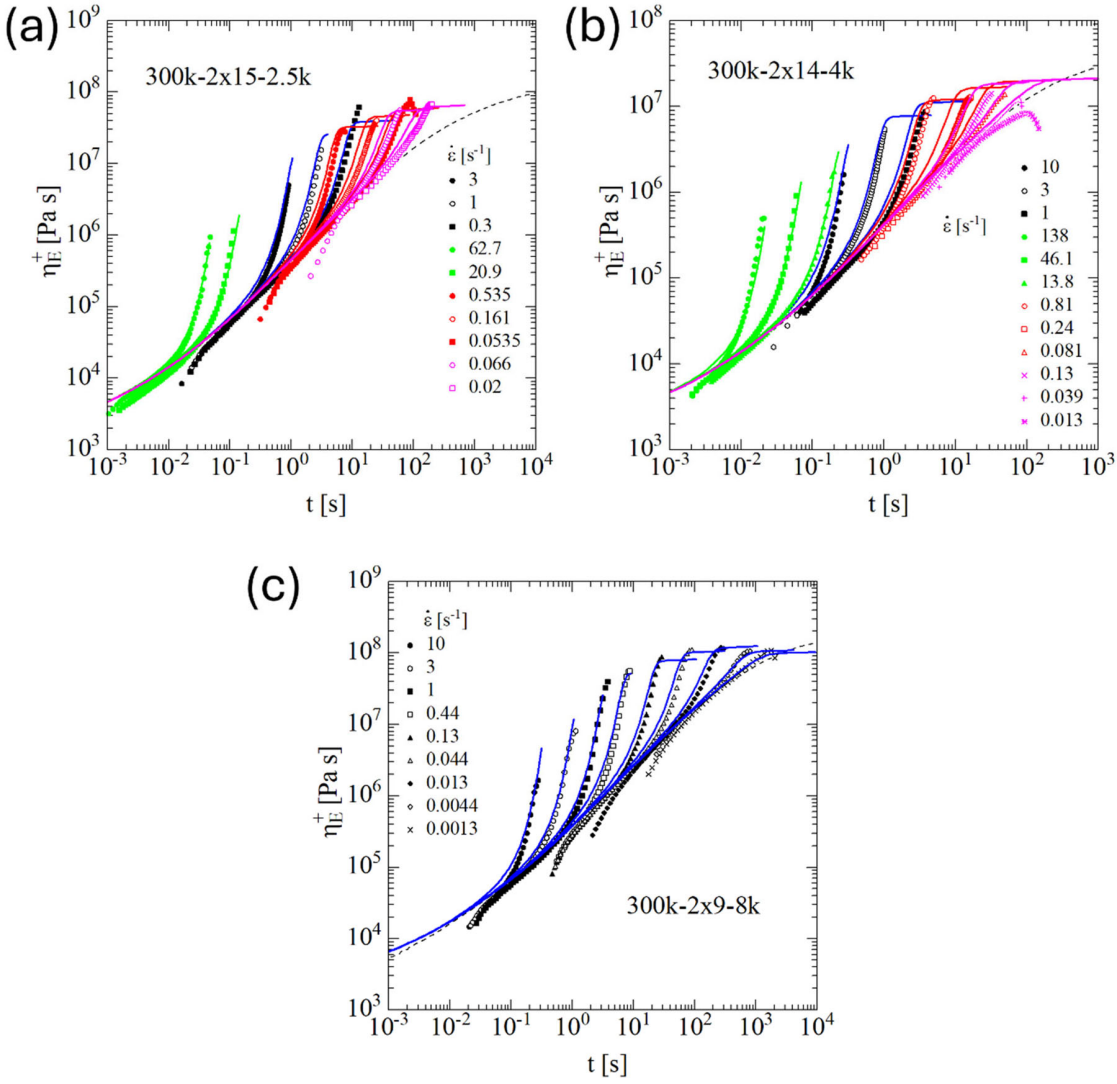
Elongational start‐up viscosity $\eta_{E}^{+}\left(t\right)$ of pom‐poms (a) 300k‐2 × 15‐2.5k, (b) 300k‐2 × 14‐4k, and (c) 300k‐2 × 14‐8k. Experimental data (symbols) measured at 130°C (green), 140°C (black), 160°C (red), and 180°C (pink), and time‐temperature shifted to 140°C. Lines are predictions of the ERS model.

In Figure 3, the elongational start‐up viscosity data (symbols) of pom‐poms 300k‐2 × 15‐2.5k, 300k‐2 × 14‐4k, and 300k‐2 × 14‐8k measured at temperatures between 130°C and 180°C and time‐temperature shifted to 140°C are presented. Except for the lowest strain rates investigated for 300k‐2 × 14‐4k, agreement with predictions of the ESR model (continuous lines) with stress tensor Equation (1) and evolution Equation (3) is obtained. At low elongation rates and high Hencky strains, a transition to a steady‐state elongational viscosity is observed in the experimental data, which is supported by the predictions of the ERS model. At high elongation rates, fracture is observed experimentally and predicted by the fracture Equation (8).

**FIGURE 4:**
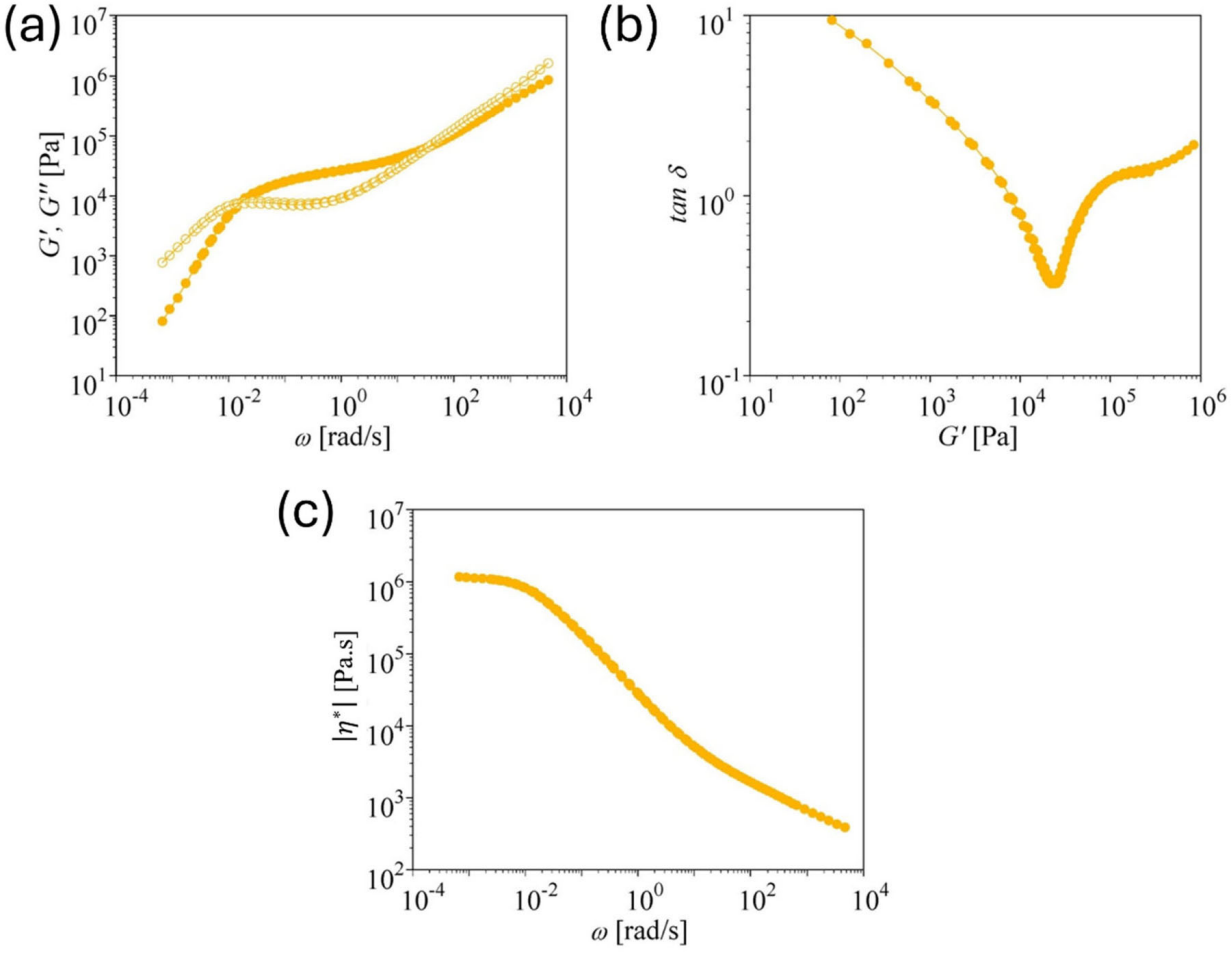
(a) Storage modulus *G*′ and loss modulus *G*″ as a function of frequency *ω* for pom‐pom 280k‐2×30‐7k. (b) Loss tangent δ as a function of *G*′. (c) Absolute value of the complex shear viscosity $|\eta^{*}\left(\omega \right)|$.

**FIGURE 5:**
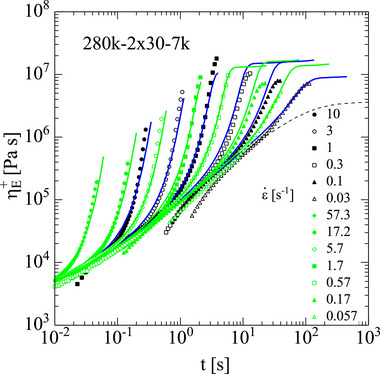
Elongational start‐up viscosity $\eta_{E}^{+}\left(t\right)$ of pom‐pom 280k‐2 × 30‐7k. Experimental data (symbols) measured at 130°C (green) and 140°C (black), and time‐temperature shifted to 140°C. Lines are predictions of the ERS model.

The master curves of storage and loss modulus, the loss tangent δ, and the absolute value of the complex shear viscosity $\eta^{*}\left(\omega \right)$ of pom‐pom 280k‐2 × 30‐7k are shown in Figure 4. Due to the strong dilution of the backbone by 30 side arms of *M_w,a_* = 7 kg mol^−1^, the plateau modulus as determined from Figure 4b is reduced to $G_{N}^{0}$ = 2.8 10^4^ Pa, indicating an effective fraction of the backbone of φ=0.33 (Table 3). Consequently, pom‐pom 280k‐2 × 30‐7k shows strong transient strain hardening with tensile stress growth coefficients $\eta_{E}^{+}\left(t\right)$ at larger Hencky strains reaching values significantly above the zero‐elongation‐rate viscosity $\eta_{E}^{0}$ *=* 3$\eta_{0}$. Again, as shown in Figure 5, the elongational start‐up viscosity is well described by the ERS model using the Rouse time calculated from Equation (4).

**FIGURE 6:**
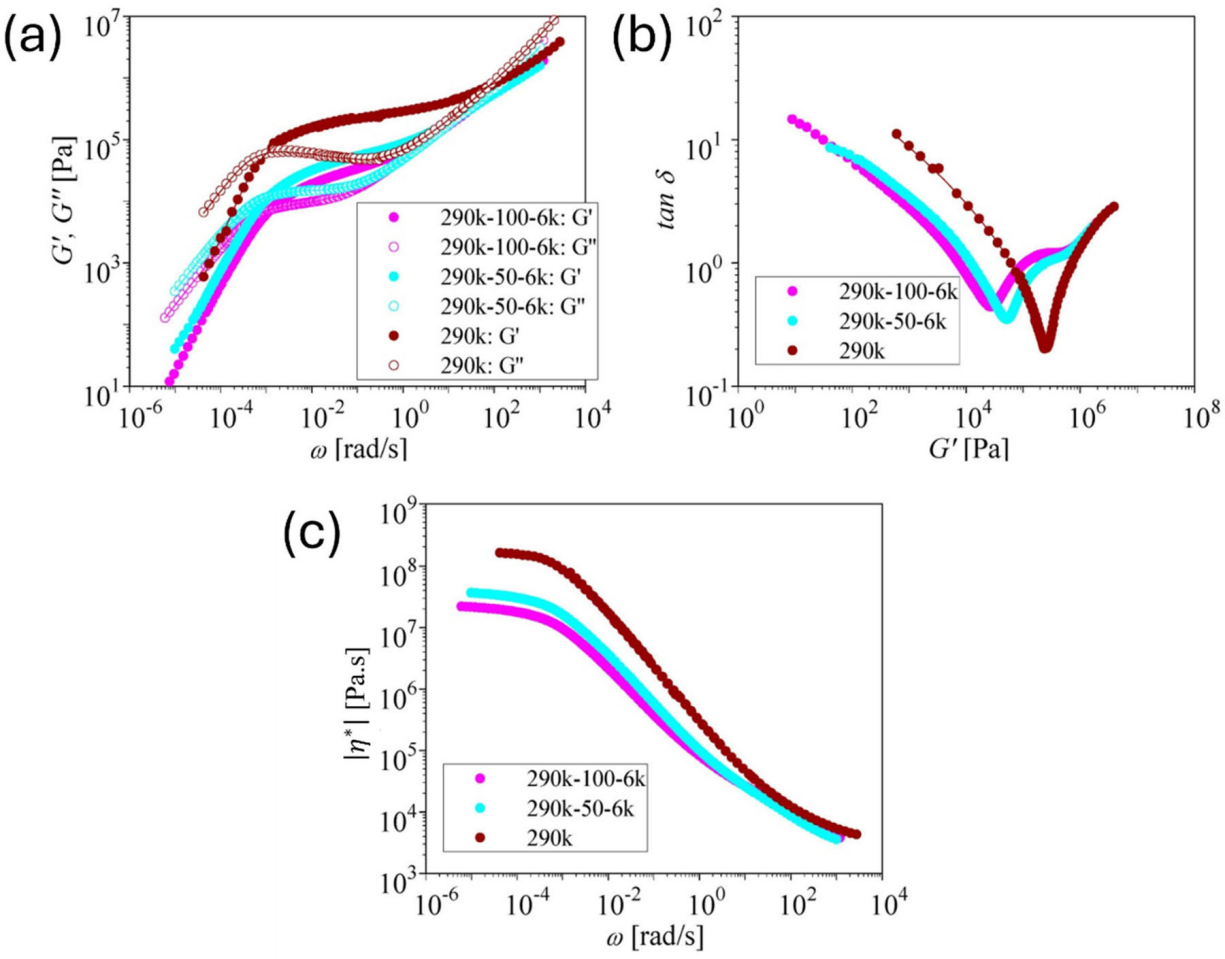
(a) Storage modulus *G*′ and loss modulus *G*″ as a function of frequency *ω* for linear PS290k as well as combs 290k‐50‐6k and 290k‐100‐6k at a reference temperature of 140°C. (b) Loss tangent δ as a function of *G*′. (c) Absolute value complex shear viscosity |$\eta^{*}\left(\omega \right)|$.

The LVE characterization of PS290k, as well as the combs 290k‐50‐6k and 290k‐100‐6k with entangled backbone of M_w,b_ = 290 kg/mol and 50 and 100 unentangled side arms of M_w,a_ = 6 kg/mol (Table 1) is presented in Figure 6. With increasing dilution of the backbone, the plateau modulus decreases (Figure 6b and Table 3).

**FIGURE 7:**
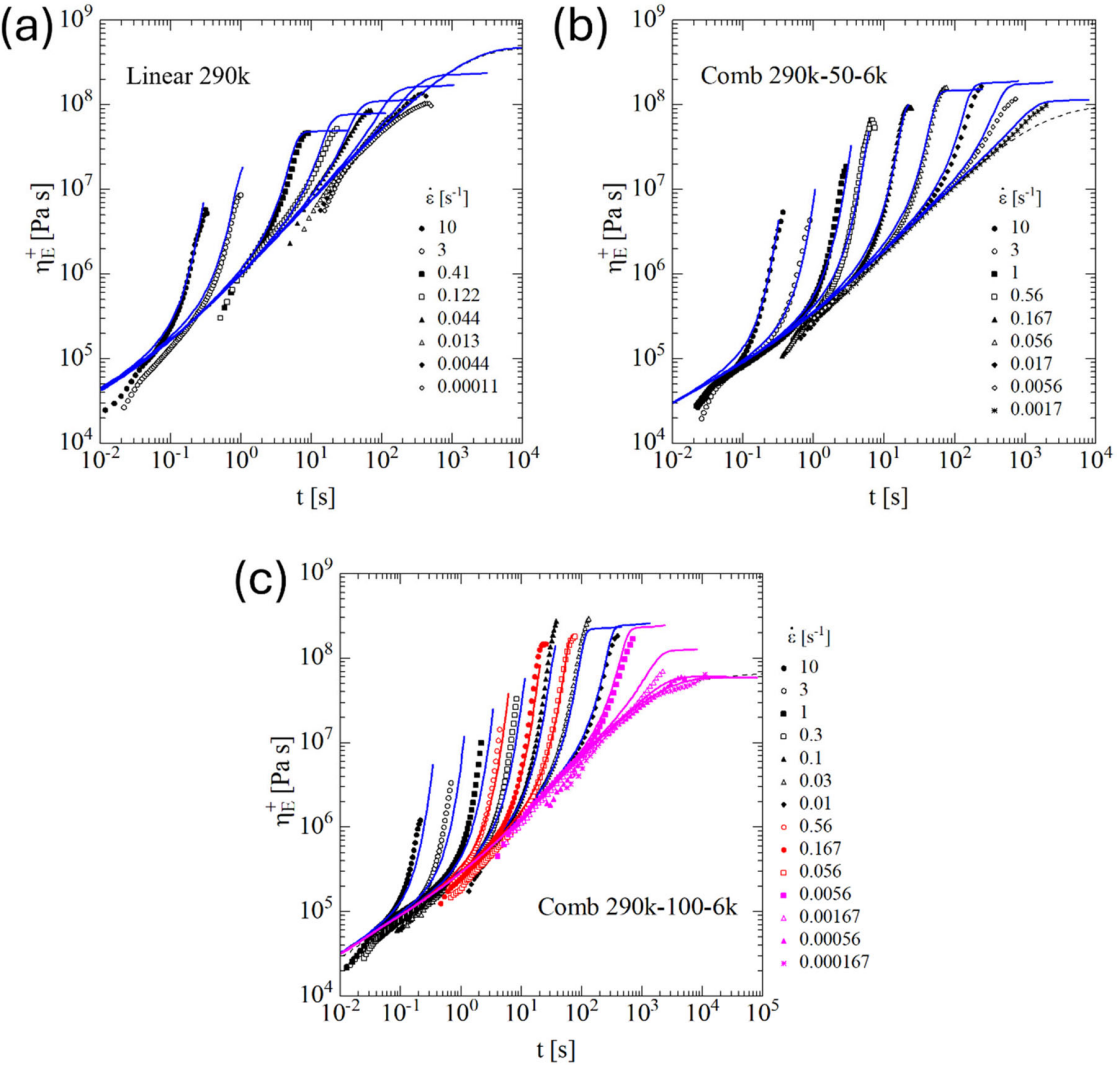
Elongational start‐up viscosity $\eta_{E}^{+}\left(t\right)$ of (a) linear PS290k, (b) comb 290k‐50‐6k, and (c) 290k‐100‐6k at 140°C. Experimental data (symbols) of 290k‐100‐6k measured at 140°C (black), 160°C (red), and 180°C (pink), and time‐temperature shifted to 140°C. Lines are predictions of the ERS model.

The elongational start‐up viscosity data (symbols) of linear PS290k and combs 290k‐50‐6k and 290k‐100‐6k measured at temperatures between 140°C and 180°C and time‐temperature shifted to 140°C are presented in Figure 7. The data indicate a transition to a steady‐state elongational viscosity at low elongation rates and high strains, while fracture is observed at higher strain rates. Predictions of the ERS model are in agreement with experimental evidence.

**FIGURE 8:**
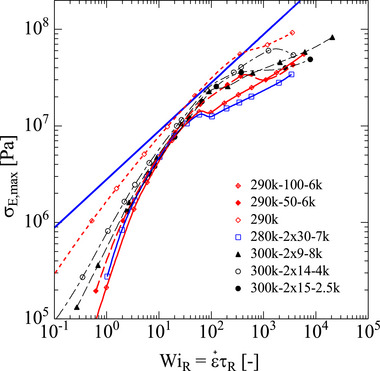
Maximal elongational stress $\sigma_{E,\max}$ of pom‐poms and combs as a function of Weissenberg number $Wi_{R}=\dot{\varepsilon }\;\tau_{R}$. Lines are calculated by use of the ERS model. We use symbols to show the calculated values of the ERS model at the strain rates we investigated experimentally to highlight the experimentally accessible window. The straight solid blue line indicates the universal relation $\sigma_{E}≅5G_{N,\mathrm{PS}\;}^{0}\sqrt{5Wi_{R}}$ with $G_{N,\mathrm{PS}\;}^{0}=250\;\mathrm{kPa}\;$.

Figure 8 shows the maximal value of the elongational stress $\sigma_{E,\;\max}$ corresponding to the steady‐state elongational $\sigma_{E}$ or the maximal elongational stress reached in the case of fracture, as a function of Weissenberg number $Wi_{R}=\dot{\varepsilon }\;\tau_{R}$ for the model polymers considered. Lines are calculated by use of the ERS model, and symbols specify the calculated values at the experimentally investigated strain rates. Because model and experimental data agree largely, the symbols also give an indication of the maximal elongational stresses reached experimentally. The straight solid blue line indicates the limiting steady‐state elongational stress $\sigma_{E}≅5G_{N,\mathrm{PS}\;}^{0}\sqrt{5Wi_{R}}$ expected at high elongation rates according to the universal relation of Equation (5). As seen in Figure 8, the elongational stress of the differently diluted polymer systems converges with increasing Weissenberg number and tends to reach the limiting elongational stress in agreement with the universal relation, i.e. at high Weissenberg numbers $Wi_{R}$ the elongational stress is independent of the amount of dilution. However, for the strongly diluted samples 290k‐100‐6k and 280k‐2×30‐7k, fracture, e.g. breaking of covalent bonds occurs before the predicted limiting stress is reached as seen by the kink in the slope. We note that the universal relation of Equation (5) was also shown to agree well with data of Shahid et al. [50, 51] on blends of 10% of a high molar mass polystyrene PS820 (monodisperse PS with 820 kg/mol) with 90% matrix styrene with molar masses of 9 kg/mol (oligomeric styrene), 23 and 34 kg/mol (marginally entangled PS, but below M_c_), and 73 kg/mol (well entangled PS), respectively, which all show the same steady‐state elongational viscosity at sufficiently large elongation rates. Therefore, even in bidisperse melts, if the difference of the molar masses of the two components is large enough such that the Rouse time of the low molar mass component is much smaller than the Rouse time of the high molar mass component and therefore at the elongation rate investigated, the low molar mass component remains unstretched and acts solely as a diluent, the steady‐state elongational stress $\sigma_{E}$ is fully determined by the high molar mass component, irrespective of the molar mass of the polymeric matrix.

**FIGURE 9:**
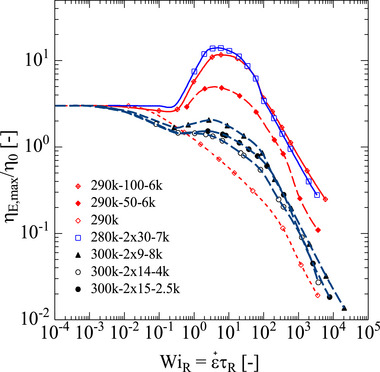
Normalized elongational viscosity $\eta_{E,\max}/\eta_{0}$ of pom‐poms and combs as a function of Weissenberg number $Wi_{R}=\dot{\varepsilon }\tau_{R}$. Lines are calculated by use of the ERS model. We use symbols to show the calculated values of the ERS model at the strain rates we investigated experimentally to highlight the experimentally accessible window.

**FIGURE 10:**
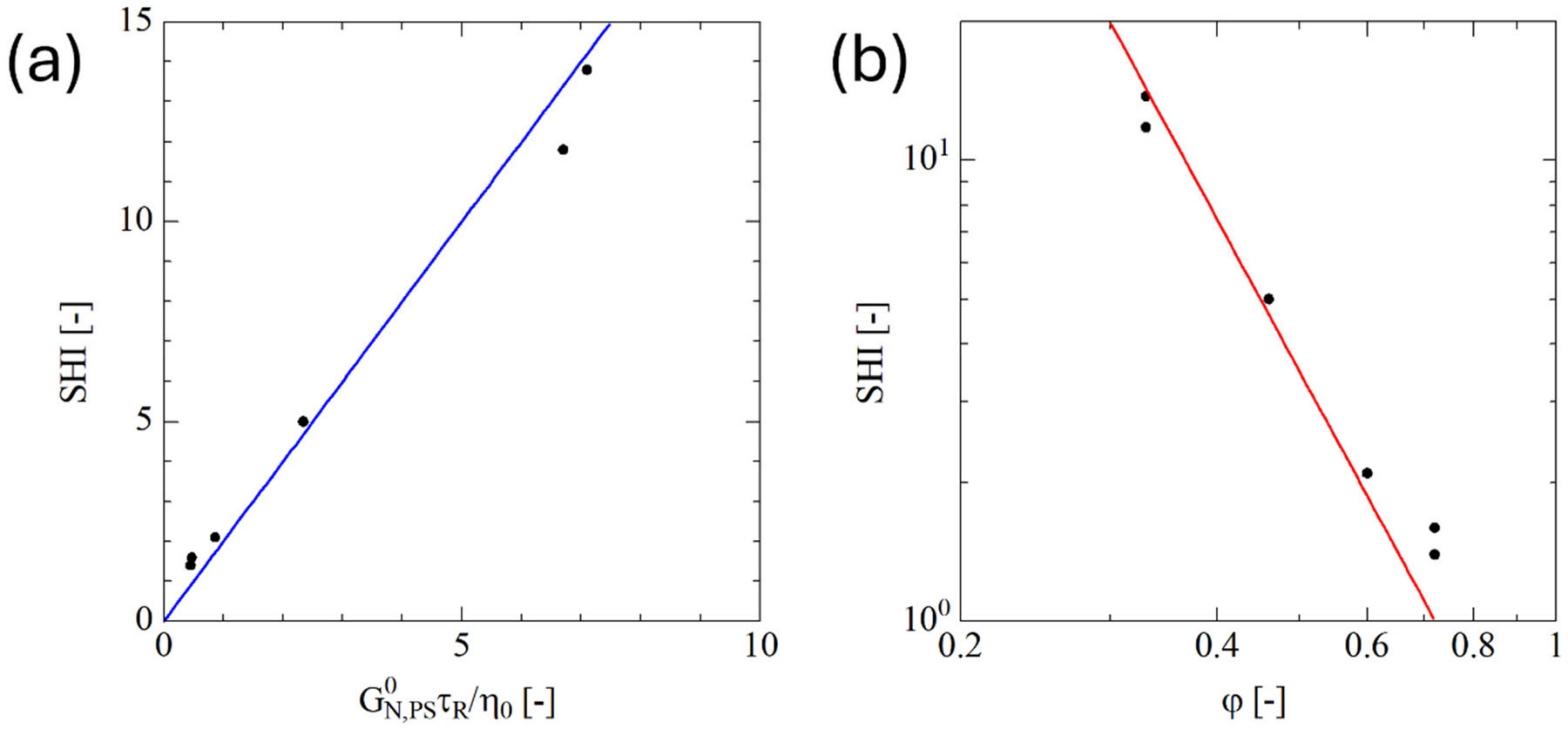
(a) SHI as a function of $\;G_{N,PS\;}^{0}\tau_{R}/\eta_{0}$. Solid blue line indicates $\;\mathrm{SHI}\;=2\times G_{N,\mathrm{PS}\;}^{0}\tau_{R}/\eta_{0}$. (b) SHI as a function of backbone fraction φ for $M_{w,b}$ = 290 kg/mol. Solid red line indicates $\mathrm{SHI}\;=\frac{1}{3}\;\varphi^{-3.4}$. Linear regression reaches SHI=1 for φ = 0.724.

The maximal value of the normalized elongational viscosity $\eta_{E,\max}/\eta_{0}$ corresponding to the steady‐state elongational viscosity or the maximal elongational viscosity reached in the case of fracture, as a function of Weissenberg number $Wi_{R}=\dot{\varepsilon }\tau_{R}$ is presented in Figure 9. While the linear PS290k melt shows a continuously decreasing elongational viscosity, the elongational viscosity of the pom‐pom and comb systems has a similar shape: $\eta_{E,\max}/\eta_{0}$ first increases starting at $Wi_{R}\approx 0.3$ and after reaching a maximum at $3\leq Wi_{R}\leq 6$, decreases with increasing $Wi_{R}$. The maximal value of $\eta_{E,\max}/\eta_{0}$ increases with decreasing volume fraction φ. The maximal value is below the Trouton ratio of $\eta_{E}^{0}\;/\eta_{0}$ *=* 3 for pom‐poms 300k‐2 × 15‐2.5k, 300k‐2 × 14‐4k, and 300k‐2 × 9‐8k with φ=0.72 and φ=0.60, respectively. $\eta_{E,\max}/\eta_{0}$ is largest for pom‐pom 280k‐2 × 30‐7k (*SHI* = 14) and comb 290k‐100‐6k (*SHI* = 12) at φ=0.33. We take this maximal value of $\eta_{E,\max}/\eta_{0}$ as a comparative measure of strain hardening and call it the Strain Hardening Index SHI (see Schußmann et al. 2025; Wagner et al. 2025). Please note that the strain hardening index relates the extensional viscosity at a given strain rate to the zero‐shear viscosity, whereas the strain hardening factor relates the extensional viscosity to the extensional viscosity predicted by the Doi‐Edwards model at the same strain rate. According to the universal relation (5a), in the limit of sufficiently large Weissenberg numbers, the effect of dilution on the normalized elongational viscosity $\eta_{E,\max}/\eta_{0}$ depends only on the quantity $\;G_{N,PS\;}^{0}\tau_{R}/\eta_{0}$. In Figure 10a, the SHI is plotted as a function of $\;G_{N,PS\;}^{0}\tau_{R}/\eta_{0}$ and approximately a linear relation of the form $\;\mathrm{SHI}=2\times G_{N,\mathrm{PS}\;}^{0}\tau_{R}/\eta_{0}$ (solid blue line) is found. We remark that according to Equation (4), $\tau_{R}/\eta_{0}$ at constant $M_{w,b}$ increases with $\varphi^{-3.4}$. Thus, we can conclude that the SHI of pom‐poms and combs with an entangled backbone and unentangled side arms increases for constant $M_{w,b}$ with increasing dilution according to

(16) $$\mathrm{SHI}\propto \varphi^{-3.4}$$

This is a remarkable finding, as it allows us to easily tune and predict the SHI of short‐chain branched topologies. Indeed, as $M_{w,b}$ of the polymer systems considered here is in the narrow range of 280–300 kg/mol, and neglecting the effect of the comparatively small variation in $M_{w,b}$, the SHI of the 7 polymer systems considered here follows roughly the power‐law relation of Equation (16) as shown in Figure 10b.

Short side arms with $M_{w,a}\;\leq \;M_{e}/2$ lead to permanent dilution of the backbone on the time scale of the experiment, and therefore to decelerated stretch relaxation according to Equation (3) of the ERS model. In contrast, in order to assess the strain hardening of molecular topologies featuring long side arms with $M_{w,a}>\;M_{e}/2$, the effect of dynamic dilution of the backbone caused by hierarchical relaxation of the side arms has to be taken into account, see, e.g., in these references [52, 53].

**FIGURE 11:**
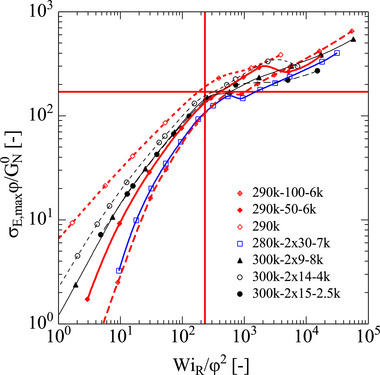
Scaled elongational stress $\sigma_{E,\max}\phi /G_{N}^{0}$ of pom‐poms and combs as a function of scaled Weissenberg number $Wi_{R}/\phi^{2}$. Symbols indicate values calculated by the ERS model at the experimentally investigated strain rates. Horizontal and vertical red lines represent the scaled critical elongational fracture stress $\frac{\sigma_{E,\max}\varphi }{G_{N}^{0}}≅170$ (Equation (9)) and the scaled critical Weissenberg number $\frac{Wi_{R}}{\varphi^{2}}≅231$ (Equation (9)), respectively.

The scaled elongational stress $\sigma_{E,\max}\phi /G_{N}^{0}$ of pom‐poms and combs as a function of scaled Weissenberg number $Wi_{R}/\phi^{2}$ is presented in Figure 11. Symbols specify the calculated values by the ERS model at the experimentally investigated strain rates. According to the fracture criterion of Equation (7), fracture is expected to occur at and above the scaled critical elongational fracture stress $\sigma_{c}\varphi /G_{N}^{0}≅170$ (Equation 9) indicated by the horizontal red line, and to the right of the scaled critical Weissenberg number $Wi_{\mathrm{Rc}}/\varphi^{2}≅231$ (Equation 10) is indicated by the vertical red line. A marked change of slope or even kinks $\sigma_{E,\max}\phi /G_{N}^{0}$ are observed above the critical Weissenberg number $Wi_{\mathrm{Rc}}$ caused by a brittle fracture of the filament. This is in agreement with the fracture data of linear PS melts and solutions analyzed by Wagner et al. [46].

## 3 Conclusions

Pom‐poms and combs with entangled backbone chains but formally unentangled side arms can be considered as quasi‐linear long chain/short chain polymer blends, consisting of backbone chains which are diluted by the side arms. When the loss angle δ is plotted as a function of the storage modulus *G*′, they feature only one minimum with tanδ<1 indicating the rubbery plateau $G_{N}^{0}$ created by the entangled backbone chains. The location of the minimum and therefore the value of the plateau modulus shifts to smaller *G′* values with increasing dilution of the backbone by the side arms. The elongational rheology of these systems can be described by the Enhanced Relaxation of Stretch (ERS) model [31], which was originally developed for linear polymers diluted by short and unentangled linear polymer chains and oligomers of the same chemistry. The steady‐state elongational stress of the pom‐poms and combs with entangled backbone chains but unentangled side arms was found to converge with increasing Weissenberg number and to become largely independent of the amount of dilution, if not preceded by fracture of covalent bonds. This agrees with the universal relation of Equation (5), predicting that the steady‐state elongational stress at high elongation rates is independent of dilution and depends only on the plateau modulus of the undiluted polymer melt and the Weissenberg number.

The maximal value of the reduced elongational viscosity $\eta_{E,\max}/\eta_{0}$ increases with increasing dilution of the backbone by the side arms and can be considered as a comparative measure of strain hardening called the strain hardening index (SHI). This is because the stretch relaxation is reduced by dilution, leading to higher chain stretching. The comparison of the elongational viscosity data of the pom‐poms (with one branching point only at each end of the backbone) versus that of the combs (with several branching points along the backbone chain) suggests that the location of the unentangled side arms does not result in significant differences in the elongational viscosity: Pom‐pom 280k‐2×30‐7k and comb 290k‐100‐6k with the same backbone volume fraction of φ=0.33 show nearly the same normalized elongational viscosity $\eta_{E,\max}/\eta_{0}$ as a function of Weissenberg number $Wi_{R}=\dot{\varepsilon }\tau_{R}$. Furthermore, we find that for comb or pom‐pom systems, the backbone volume fraction is the key criterion to predict the strain hardening index (SHI): At constant *M_w,b_*, the SHI follows a power law of the form $SHI\propto \varphi^{-3.4}$. The SHI increases with increasing dilution.

At higher strain rates, brittle fracture is observed, which is well described by the fracture criterion for linear melts and polymer solutions [46], confirming the effect of self‐dilution of the backbone chains of pom‐poms and combs by unentangled side arms.

## Conflicts of Interest

The authors declare no conflict of interest.

## Data Availability

The data that support the findings of this study are available from the corresponding author upon reasonable request.
